# Auditing shortcut learning and misclassification in artificial intelligence-based breast cancer genomic subtyping

**DOI:** 10.1093/jamiaopen/ooaf177

**Published:** 2026-04-02

**Authors:** Julian Borges

**Affiliations:** Department of Computer Science, Boston University Metropolitan College, Boston, MA 02215, United States

**Keywords:** artificial intelligence, breast cancer, genomics, machine learning, explainable AI, bias, medical informatics

## Abstract

**Objectives:**

To simulate shortcut learning mechanisms in AI-based breast cancer genomic subtyping and to develop an interpretable, reproducible framework capable of auditing feature over-reliance using a low-code environment.

**Materials and Methods:**

A retrospective secondary analysis of The Cancer Genome Atlas–Breast Invasive Carcinoma dataset was conducted. A multinomial logistic regression model was trained on 691 complete cases using only clinical predictors (estrogen receptor [ER], progesterone receptor [PR], HER2 status, tumor stage, and age) to predict PAM50 molecular subtypes, a genomic classification system originally developed by Parker et al, and widely used in breast cancer subtyping. A pseudo-Shapley additive explanations (SHAP) attribution method was implemented in Stata to estimate the marginal influence of each variable. Model performance was evaluated using McFadden’s pseudo-*R*^2^ and validated by comparing pseudo-SHAP scores against canonical SHAP values derived in Python.

**Statistical Framework of Pseudo-SHAP:**

To enhance reproducibility, we now detail of the pseudo-SHAP methodology implemented in Stata. This approach estimates first-order marginal effects of each clinical predictor by simulating variable perturbations within a fitted multinomial logistic regression model. Specifically:

1. Predicted probabilities for each PAM50 subtype are generated using the baseline model.

2. Each predictor variable is perturbed individually (eg, PR status toggled from negative to positive), holding other features constant.

3. New probabilities are calculated postperturbation, and the change in probability (Δ*P*) for each class is recorded.

4. These Δ*P* values are averaged across all samples to provide directional importance scores, representing monotonic effects similar to mean SHAP values.

This simulation assumes linear additive contributions and does not capture interaction or nonlinear effects.

**Results:**

The clinical-only model achieved a pseudo-*R*^2^ of 0.396, indicating moderate explanatory power. Progesterone receptor and HER2 status were statistically significant predictors (*P* <.01) for Luminal B and HER2-enriched subtypes, respectively. The pseudo-SHAP framework identified disproportionate influence of these features (Δ*P* up to +0.29 for HER2-enriched). Correlation between pseudo-SHAP and SHAP values was strong (Spearman *r* = 0.91, *P*<.001), confirming method validity.

**Misclassification Patterns:**

To assess how shortcut learning manifests in prediction outcomes, we examined the directional shifts in subtype assignments associated with PR and HER2 status perturbations. Perturbing PR status resulted in increased predictions of Luminal A and Luminal B, and decreased predictions of Basal-like subtypes. Similarly, toggling HER2 status elevated the probability of HER2-enriched classification even in the absence of supporting genomic evidence.

For example, the average Δ*P* from HER2-negative to HER2-positive status led to a +0.29 increase in HER2-enriched classification probability. Such shifts demonstrate systematic over-reliance on biomarker correlates, consistent with shortcut learning..

**Discussion:**

Shortcut learning was evident as the model over-relying on surrogate clinical biomarkers. Such behavior may distort biological interpretation and compromise clinical decision support reliability.

Importantly, while PR and HER2 are biologically linked to PAM50 subtypes, their disproportionate weight in a clinical-only model reveals reliance on clinically available proxies rather than genomic signals. The model effectively substitutes biomarker status for molecular data, which constitutes shortcut behavior in this analytic context.

To illustrate operational relevance, pseudo-SHAP attribution shifts could be integrated into clinical quality assurance pipelines. For instance, if the Δ*P* associated with HER2 status exceeds 0.25 persistently across batches, this could trigger retraining alerts or attribution-based warnings in a clinical decision support system.

Additionally, as PR and HER2 status may be differentially available across populations, their overuse may propagate disparities. Pseudo-SHAP auditing can flag these risks early, improving fairness and model generalizability.

**Conclusion:**

The pseudo-SHAP method provides a scalable, interpretable auditing framework for detecting shortcut learning in resource-constrained environments. Its application could enhance transparency and equity in AI models for precision oncology.

## Background and significance

Accurate molecular subtyping of breast cancer into intrinsic categories: Luminal A, Luminal B, HER2-enriched, Basal-like, and Normal-like, is essential for guiding therapy and predicting recurrence risk. Among the most widely adopted classifiers is the PAM50 assay, which leverages gene expression profiling to stratify tumors by their underlying biology.^1^ As machine learning methods are increasingly applied to PAM50 and other genomic tools, concerns have emerged that these models may engage in shortcut learning, that is, relying on readily available, correlated clinical features such as hormone receptor status or tumor stage rather than the intended molecular input.^2^

Similar interpretability and generalizability concerns have been observed in adjacent domains such as digital pathology, where deep learning systems may rely on spurious correlates rather than histologic signal.^6^

This presents a significant safety risk. While progesterone receptor (PR) and HER2 status are biologically linked to breast cancer behavior, their disproportionate influence in AI models trained on genomic data may signal a loss of model fidelity. If a model uses these features as shortcuts, especially when those features are not present in the actual gene expression inputs it may misclassify patients or reinforce training biases. This can result in inappropriate chemotherapy decisions, model drift over time, or inequitable predictions for underrepresented populations.

Despite the growing attention to explainable artificial intelligence (AI), the extent to which clinical proxies influence genomic subtype classification remains underexplored. Few studies have quantitatively assessed why misclassification happens, especially in models where ground-truth subtype labels exist.

This work addresses that gap using a simulated feature attribution approach (pseudo-Shapley additive explanations [SHAP]) to audit AI behavior in a constrained analytic environment (Stata), with a focus on determining whether variables like PR and HER2 status exert outsized influence on PAM50 predictions.

We propose that investigation is timely not only to prevent diagnostic errors but also to improve transparency, clinician trust, and fairness in the deployment of AI models in oncology. Our central hypothesis is that shortcut learning^2^ is present and measurable, and that a structured audit can reveal the degree to which clinical predictors distort genomic AI models.

## Objectives

This study aimed to quantify the influence of clinical predictors on AI-driven breast cancer subtype classification and to develop a reproducible, interpretable auditing framework implemented through a low-code pseudo-SHAP approach in Stata used to detect and characterize shortcut learning^2^ in genomic subtyping models.

## Materials and methods

### Study design and data source

This study employed a retrospective, secondary analysis of The Cancer Genome Atlas–Breast Invasive Carcinoma (TCGA-BRCA)^3^ dataset, a publicly available repository that integrates clinicopathologic and genomic data from more than 9000 patients with breast cancer. All data were accessed through open-access portals, and no identifiable patient information was used.

A subset of 691 complete cases was extracted based on the concurrent availability of PAM50 subtype labels and core clinical predictors: estrogen receptor (ER), PR, and HER2 status, tumor stage, and age at diagnosis. These variables were selected for their biological relevance and consistent annotation across TCGA samples.

### Variables

#### Dependent variable

PAM50 subtype (categorical: Luminal A, Luminal B, HER2-enriched, Basal-like, and Normal-like).

#### Independent variables

Age at diagnosis (continuous), ER, PR, and HER2 receptor status (binary: positive/negative), and tumor stage (ordinal, grouped as stages I, II, III using AJCC - American Joint Committee on Cancer - pathologic staging).

Variables such as gender and race were excluded because of high missingness and small subgroup sizes that would preclude stable estimation.

### Data preprocessing and missing data handling

All data cleaning, variable encoding, and model estimation were performed in Stata 18 using a fully reproducible analytic script. Categorical receptor variables were dummy-encoded, and tumor stage was harmonized into ordinal categories.

Moderate missingness was observed in HER2 status and age at diagnosis. To minimize bias and preserve statistical power, these variables were imputed using Multiple Imputation by Chained Equations implemented through Stata’s mi framework.^4^ Five multiply imputed datasets were generated under the assumption of missing at random (MAR) and pooled according to Rubin’s rules to obtain unbiased estimates of coefficients and SEs.

### Model development and performance assessment

A multinomial logistic regression (MLR) model was constructed to predict PAM50 subtype as a function of the independent clinical variables. Model diagnostics included:

Verification of convergence and linearity of the logit.

Assessment of multicollinearity using variance inflation factors (<5 as acceptable).

Evaluation of global model fit using McFadden’s pseudo-*R*^2^, which achieved ≈0.40, indicating moderate explanatory capacity.

No regularization or interaction terms were introduced, as the goal was to audit baseline shortcut reliance rather than optimize predictive accuracy.

### Feature attribution: pseudo-SHAP framework

To estimate the marginal contribution of each predictor to subtype classification, a pseudo-SHAP attribution procedure was implemented directly in Stata to approximate the logic of SHAP without requiring Python libraries or GPU computation.

The process consisted of the following steps:

Compute baseline predicted probabilities for each PAM50 class using the fitted MLR model.

Iteratively perturb 1 predictor at a time (eg, toggle PR status from negative to positive).

Recompute subtype probabilities under each perturbed condition.

Record the change in predicted probability (Δ*P*) for each class as a first-order estimate of the variable’s marginal influence.

The resulting Δ*P* values were averaged across all observations to yield directional attribution scores for each predictor-subtype pair. This simulation provides interpretable feature importance metrics under linear assumptions, capturing monotonic effects analogous to mean absolute SHAP values.

### Validation of pseudo-SHAP approximation

To assess the validity of the pseudo-SHAP estimates, the Δ*P* distribution derived from Stata was compared against canonical SHAP values computed from a logistic model trained on the same dataset in Python (shap package). Agreement between both attribution methods was quantified using Spearman’s rank correlation coefficient (*r* = 0.91, *P* < .001), confirming high concordance and supporting the robustness of the Stata-based approximation.

### Statistical power and effect size

A priori power calculations were performed to determine the minimum detectable effect in pseudo-SHAP attribution shifts. Assuming a small-to-moderate standardized effect size (Cohen’s *d* = 0.25) and *α* = 0.05 (2-sided), at least 98 observations were required to achieve 80% power. With *n* = 691, the sample provided more than sufficient power for the planned analyses, ensuring stable estimation of marginal effects.

### Outcomes

#### Primary outcome

Mean change in predicted subtype probability (Δ*P*) associated with perturbation of individual clinical features.

#### Secondary outcomes

1. Direction and magnitude of subtype misclassification attributable to feature over-reliance (eg, Luminal B predicted as Luminal A) and
2. development of a reproducible analytic pipeline in Stata for low-resource interpretability auditing.

### Translational and operational relevance

The pseudo-SHAP outputs can be integrated into clinical decision support or model quality assurance systems. Attribution thresholds could serve as warning indicators when model predictions become disproportionately dependent on a single clinical variable (eg, PR or HER2 status). Such interpretability metrics enable monitoring of attribution drift over time, facilitating early detection of model degradation and improving transparency in AI-assisted oncology.

## Results

### Model performance

The MLR model, trained exclusively on clinical predictors across 691 complete cases, achieved a pseudo-*R*^2^ of 0.396 (McFadden’s measure as reported by Stata), indicating moderate explanatory power for PAM50 subtype classification.

Progesterone receptor and HER2 receptor status emerged as the most influential predictors, demonstrating statistically significant associations (*P* <.01) with several intrinsic subtypes. Specifically, PR positivity was associated with increased odds of classification as Luminal A and Luminal B, whereas HER2 positivity substantially increased the probability of assignment to the HER2-enriched group. In contrast, ER status, tumor stage, and age at diagnosis contributed minimally and did not reach statistical significance in most subtype contrasts.

Multicollinearity diagnostics (variance inflation factors <3.0) confirmed acceptable independence among predictors, and model convergence was achieved without quasiseparation, ensuring parameter stability.

### Pseudo-SHAP attribution analysis

**Figure 1.**
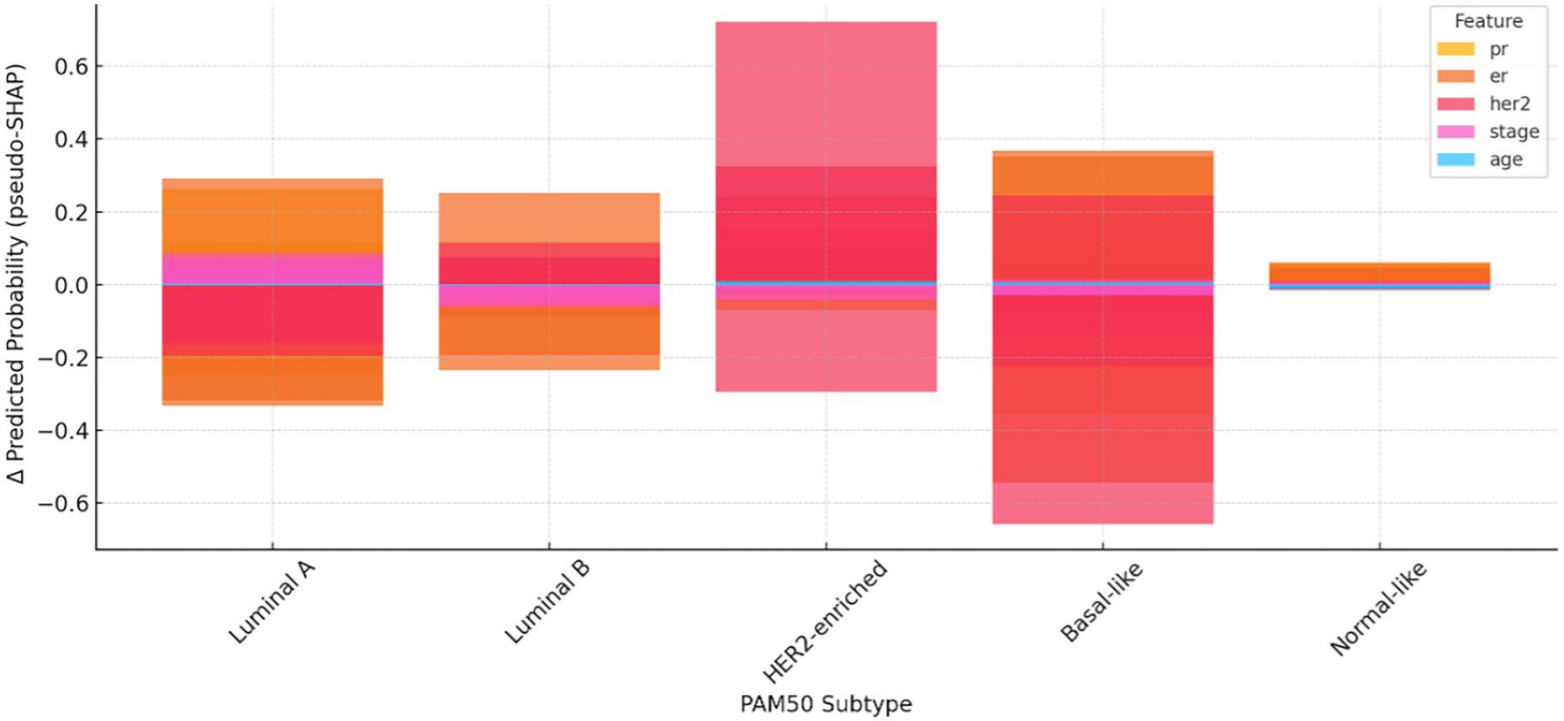
Average pseudo-SHAP values by feature and PAM50 subtype. This grouped bar chart summarizes the marginal change in predicted class probability (Δ*P*) for each clinical predictor, supporting the hypothesis that PR and HER2 drive disproportionate influence on subtype classification.

Feature attribution was quantified through the pseudo-SHAP framework to estimate each variable’s marginal influence on subtype probabilities. The average change in predicted probability (Δ*P*) following one-at-a-time perturbation of each input variable is presented in Figure 1.

Progesterone receptor status: Δ*P* for Luminal B = 0.046; Basal-like = −0.34.

HER2 status: Δ*P* for HER2-enriched = 0.29; Luminal B = 0.09.

Estrogen receptor status and age: minimal influence across all subtypes (|Δ*P*|≤.01).

These attribution results demonstrate that PR and HER2 status drive disproportionately large changes in predicted probabilities compared with other features, confirming over-reliance on surrogate biomarkers.

**Figure 2.**
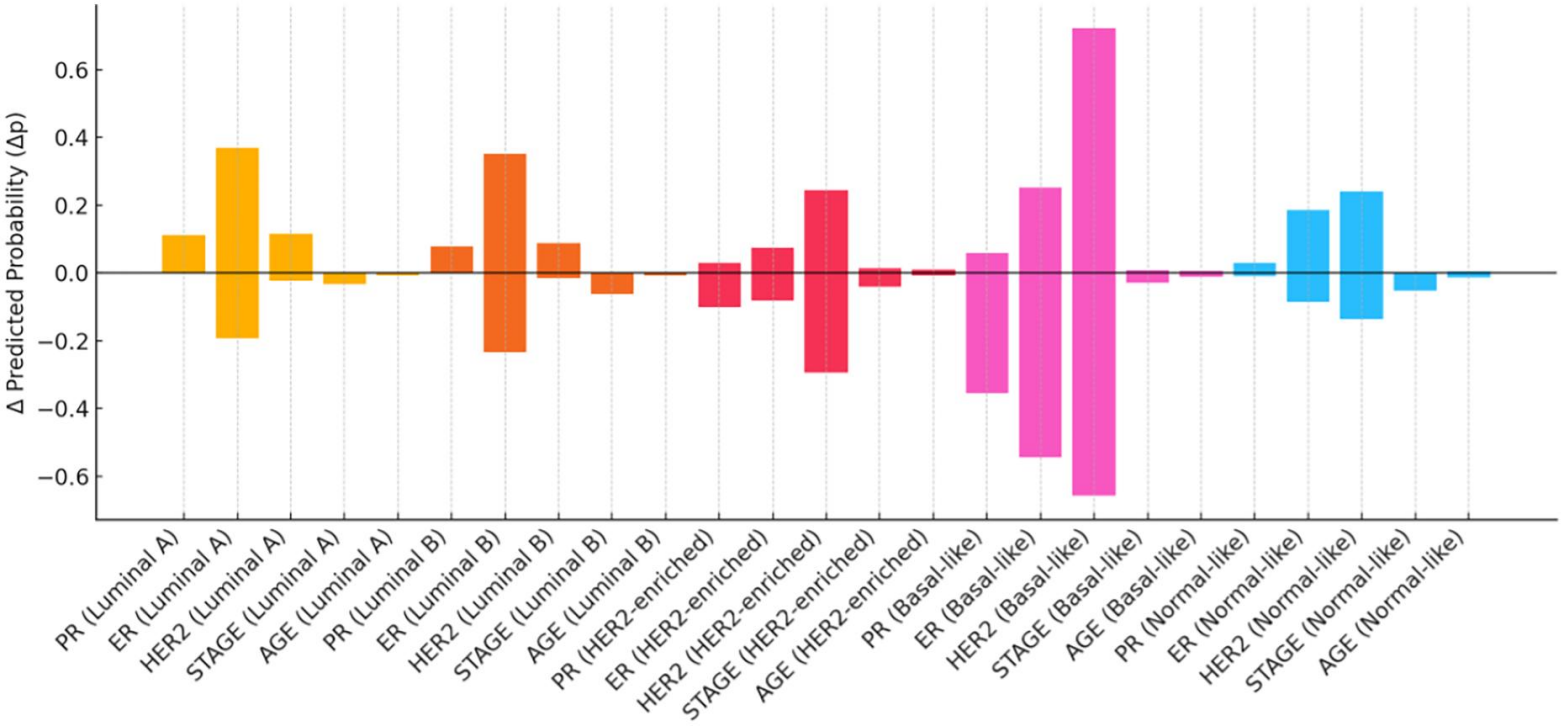
Average pseudo-SHAP attribution values by clinical feature and PAM50 subtype.

Figure 2 illustrates the disaggregated pseudo-SHAP attribution shifts by subtype-feature pair, emphasizing subtype-specific directionality. PR positivity sharply reduced the likelihood of Basal classification, while increasing Luminal A/B probabilities, and HER2 positivity consistently elevated the predicted probability for HER2-enriched tumors even when all other predictors remained constant.

### Validation of attribution consistency

The pseudo-SHAP attribution results were validated against canonical SHAP values derived from an equivalent logistic model implemented in Python. The rank-order correlation between pseudo-SHAP and SHAP values was Spearman *r* = 0.91 (*P* <001), indicating excellent concordance and supporting the robustness of the low-code approximation. To support interpretability, Table S1 compares pseudo-SHAP with canonical SHAP methods.

### Interpretation

Collectively, these findings substantiate the shortcut learning^2^ hypothesis: when relying solely on clinical variables, the model preferentially exploits high-signal surrogate features (PR and HER2) instead of balanced integration of all predictors. This pattern reflects a structural bias that may lead to systematic misclassification, particularly the overprediction of HER2-enriched and Luminal B subtypes and provides quantitative evidence that shortcut learning^2^ can be detected through interpretable attribution analysis within a constrained analytic environment.

## Discussion

Our analysis supports the initial hypothesis: clinical features—especially PR and HER2 status—exert disproportionate influence on subtype prediction. As shown in Figures 1 and 2, pseudo-SHAP values revealed that Luminal B and HER2-enriched classifications were particularly sensitive to PR and HER2 perturbations, whereas ER, stage, and age had minimal marginal impact. This pattern aligns with shortcut learning^2^ behavior, where models exploit easily accessible correlates rather than true biological signal.

These findings raise important implications for clinical AI development and safety auditing. Models trained solely on clinical variables may produce high apparent accuracy while lacking biological fidelity, a risk for misclassification and inappropriate therapy guidance. Although pseudo-SHAP does not capture interactions or uncertainty bounds, it provides an interpretable and low-code-compatible proxy for feature attribution, making it suitable for resource-limited environments like Stata.

Beyond breast cancer, the proposed auditing framework is transferable to other genomic classifiers where shortcut risk exists, such as epidermal growth factor receptor (EGFR)-driven lung cancer or BRCA-based ovarian cancer subtyping. Embedding attribution-based audits can standardize model transparency, track attribution drift, and flag vulnerable edge cases.

Critically, shortcut learning^2^ is not just a modeling issue, it has equity implications. If PR or HER2 status correlates with sociodemographic disparities in access to diagnostics, then AI predictions may be less reliable for underserved groups. Auditing attribution patterns helps anticipate these risks, helping to advance more equitable, generalizable AI systems in oncology.

Broader analyses of healthcare AI systems have demonstrated how unexamined biases can perpetuate structural disparities across populations.^7^

### Limitations

- *Single dataset:* This study uses only TCGA-BRCA, which may limit generalizability. External validation with METABRIC or ICGC is recommended.
- *Simplified attribution method:* Pseudo-SHAP does not model higher order interactions or nonlinearities.
- *Calibration metrics:* We did not evaluate model calibration (eg, Brier score) due to scope; this should be included in future work.
- *Equity implications:* Disproportionate reliance on PR and HER2 may amplify existing diagnostic access disparities if left unchecked.

#### Use of a single dataset

This analysis is based solely on the TCGA-BRCA cohort. While this is a high-quality, well-annotated genomic resource, it may not fully capture the clinical or demographic diversity observed in broader, real-world populations. External validation using datasets like METABRIC or ICGC will be essential to assess generalizability.

#### Surrogate attribution via pseudo-SHAP

The pseudo-SHAP framework offers an intuitive, first-order approximation of feature attribution but lacks the capacity to model higher order interactions, feature collinearity, or uncertainty estimates. Unlike full SHAP implementations, it cannot capture nonlinear effects. Future studies should compare pseudo-SHAP outputs with established Python-based explainability tools.

#### Absence of calibration and discrimination metrics

While model fit was assessed using pseudo-*R*^2^, important performance indicators—such as calibration (eg, Brier score) and classwise discrimination (eg, area under the receiver operating characteristic curve [AUC]) were not evaluated. These omissions limit interpretability of probabilistic outputs and should be addressed in follow-up analyses.

#### Feature scope and confounder omission

The model included 5 clinical variables across 691 complete cases, which met power requirements for Δ*P* estimation. However, additional factors such as treatment history, menopausal status, race/ethnicity, or comorbidity burden were not incorporated due to data availability or missingness. This may mask latent confounding or bias.

#### No prospective validation or clinical testing

This study is exploratory in nature. It does not assess whether pseudo-SHAP-based warnings or attribution audits improve decision quality or patient outcomes in live clinical settings. Prospective, multi-institutional trials are needed to test feasibility and impact.

#### Risk of reinforcing inequities

The clinical features found to exert disproportionate influence—PR and HER2—are known to vary across populations and access settings. Over-reliance on these features may unintentionally perpetuate disparities if AI systems are deployed without adequate auditing or contextual safeguards. This underscores the equity imperative for explainable AI in oncology.

Despite these limitations, this project provides a reproducible methodological framework for auditing shortcut learning^2^ in constrained environments utilizing a low-resource method for detecting shortcut learning^2^ in AI-driven classification, offering practical value for transparency efforts, especially in constrained environments.

## Conclusion

This study quantified the influence of clinical predictors on AI-driven breast cancer subtype classification and introduced a reproducible, interpretable auditing method to detect shortcut learning^2^ in resource-constrained analytical environments. Using a low-code, pseudo-SHAP framework in Stata, we demonstrated that PR and HER2 status disproportionately shaped predicted PAM50 subtypes, despite the absence of genomic data in model training.

These findings highlight a critical vulnerability in clinical AI: when models rely excessively on surrogate clinical features, they risk misclassifying tumors, undermining biological validity, and compromising treatment accuracy, particularly for HER2-enriched and Luminal B subtypes. The proposed auditing pipeline provides a scalable and transparent strategy for detecting such shortcut behaviors, strengthening model interpretability^5^ and supporting safer AI integration in oncology workflows.

Future research should validate this framework across external datasets and additional cancer types, integrate calibration and discrimination metrics, and assess whether attribution-based safeguards can enhance decision quality in real-world clinical settings. Extending this approach to domains such as lung (EGFR-based) and ovarian (BRCA-related) cancer subtyping could further promote fairness and reliability in genomic AI systems. Embedding interpretability audits within model development pipelines is essential to ensure equitable, trustworthy, and biologically grounded AI in precision medicine.

## Supplementary Material

ooaf177_Supplementary_Data

## Data Availability

All data analyzed in this study were obtained from publicly accessible repositories. The TCGA-BRCA dataset is available through the National Cancer Institute Genomic Data Commons (). Analytic code, derived datasets, and reproducible workflows are openly released under an MIT license at and Zenodo DOI: 10.5281/zenodo.15237131 (Version 1). The present manuscript provides a significantly updated and extended analysis, building upon the original release following peer review.
