# Supplementary figures and images for: Auditing shortcut learning and misclassification in artificial intelligence-based breast cancer genomic subtyping
Source: JAMIA Open. 2026 Apr 2;9(2):ooaf177. doi: 10.1093/jamiaopen/ooaf177 (PMC13044511; doi:10.1093/jamiaopen/ooaf177)

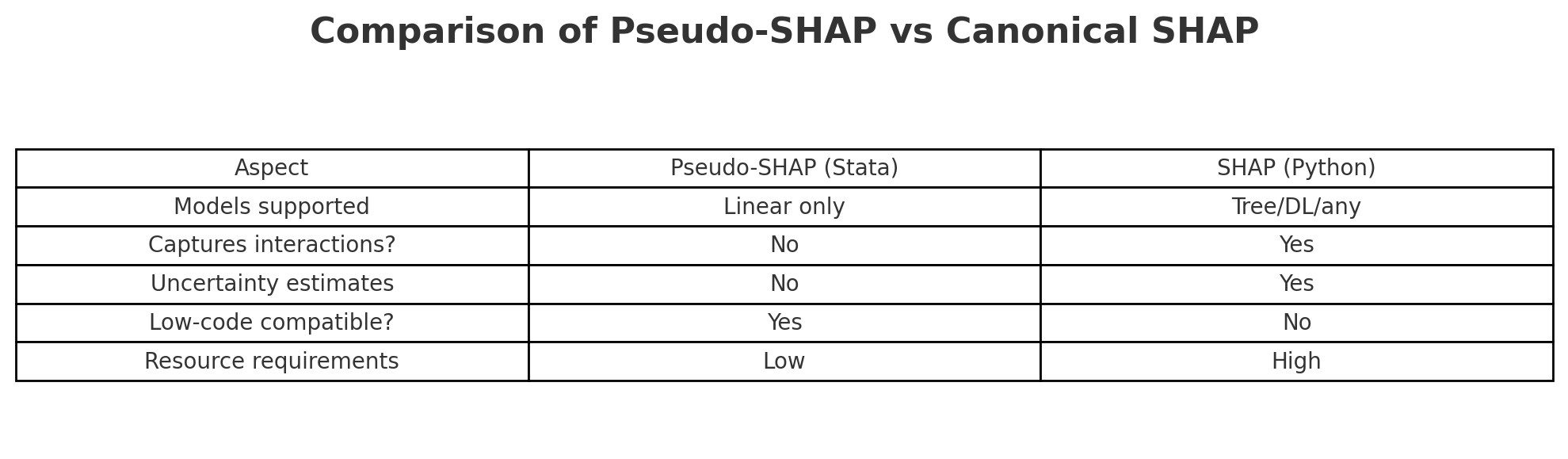

Supplement: ooaf177_Supplementary_Data [file ooaf177_supplementary_data.jpeg]
